# Ultrasensitive Electrochemical DNA Biosensor Fabrication by Coupling an Integral Multifunctional Zirconia-Reduced Graphene Oxide-Thionine Nanocomposite and Exonuclease I-Assisted Cleavage

**DOI:** 10.3389/fchem.2020.00521

**Published:** 2020-07-09

**Authors:** Zhiqiang Chen, Xueqian Liu, Dengren Liu, Fang Li, Li Wang, Shufeng Liu

**Affiliations:** ^1^Key Laboratory of Optic-Electric Sensing and Analytical Chemistry for Life Science, Ministry of Education, College of Chemistry and Molecular Engineering, Qingdao University of Science and Technology, Qingdao, China; ^2^College of Marine Science and Biological Engineering, Qingdao University of Science and Technology, Qingdao, China

**Keywords:** electrochemical DNA biosensor, reduced graphene oxide, zirconia, signal amplification, exonuclease I

## Abstract

In this work, a simple but sensitive electrochemical DNA biosensor for nucleic acid detection was developed by taking advantage of exonuclease (Exo) I-assisted cleavage for background reduction and zirconia-reduced graphene oxide-thionine (ZrO_2_-rGO-Thi) nanocomposite for integral DNA recognition, signal amplification, and reporting. The ZrO_2_-rGO nanocomposite was obtained by a one-step hydrothermal synthesis method. Then, thionine was adsorbed onto the rGO surface, *via* π-π stacking, as an excellent electrochemical probe. The biosensor fabrication is very simple, with probe DNA immobilization and hybridization recognition with the target nucleic acid. Then, the ZrO_2_-rGO-Thi nanocomposite was captured onto an electrode *via* the multicoordinative interaction of ZrO_2_ with the phosphate group on the DNA skeleton. The adsorbed abundant thionine molecules onto the ZrO_2_-rGO nanocomposite facilitated an amplified electrochemical response related with the target DNA. Since upon the interaction of the ZrO_2_-rGO-Thi nanocomposite with the probe DNA an immobilized electrode may also occur, an Exo I-assisted cleavage was combined to remove the unhybridized probe DNA for background reduction. With the current proposed strategy, the target DNA related with P53 gene could be sensitively assayed, with a wide linear detection range from 100 fM to 10 nM and an attractive low detection limit of 24 fM. Also, the developed DNA biosensor could differentiate the mismatched targets from complementary target DNA. Therefore, it offers a simple but effective biosensor fabrication strategy and is anticipated to show potential for applications in bioanalysis and medical diagnosis.

## Introduction

Highly sensitive detection of nucleic acid analytes is always pursued by researchers to accommodate the ever-increasing demands in disease diagnosis, environmental and food monitoring, and forensic identification (Debouck and Goodfellow, 1999; Liu et al., 2008; Zhang et al., 2013; Chen et al., 2019a). Polymerase chain reaction is the most commonly used method for the detection of low amounts of nucleic acids (Asiello and Baeumner, 2011; Xu et al., 2017), but the delicate temperature control by a relatively expensive thermal cycler and the complex primer design limit its wide applications, especially in some resource-constrained regions. Compared with it, biosensor technology is especially attractive for bioanalysis due to its potential advantages including economy, portability, and facile operation. Until now, various techniques such as electrochemistry, spectroscopy, surface plasmon resonance, electrochemiluminescence, and photoelectrochemistry have been well-explored for nucleic acid biosensor fabrication (Hu et al., 2014; Shi et al., 2016; Ding et al., 2017; Feng et al., 2017; Li et al., 2018). Among them, the electrochemical method possesses some inherent advantages such as simple instrumentation, signal stability, flexible operation, and easy integration and miniaturization (Tan et al., 2015; Wang et al., 2015; Liu et al., 2017). In order to upgrade the DNA detection sensitivity to satisfy the requirement of profiling trace amounts of nucleic acids, various signal amplification strategies have been explored for biosensor fabrication, for example, nuclease-based target recycling or enzyme-free DNA assembly strategies have been well-developed (Wang et al., 2014; Chen et al., 2019b). By virtue of various nucleases such as nucleic acid endonuclease, exonuclease, and polymerase, the target DNA amount could be indirectly amplified owing to target cycling or generation of target analogs. Enzyme-free nucleic acid assembly strategies such as catalytic hairpin assembly, hybridization chain reaction, and DNA-fueled target recycling are usually based on the cascade toehold-mediated strand displacement reactions for signal amplification toward target DNA recognition events (Lv et al., 2015; Ding et al., 2018; Karunanayake Mudiyanselage et al., 2018). Although these nucleases or enzyme-free DNA strategies could substantially improve the detection limit of the target DNA, the relatively complex or rigorous sequence design or the use of too much DNA fragments or various nucleases for signal amplification increases the assay cost and also the risk for error readouts. Nanomaterial or enzyme-based post-amplification means could be considered as another kind of widely used strategies for the amplified detection of nucleic acid. In these strategies, a “sandwich-type” detection mode is usually adopted with the first immobilization of capture probe DNA on the electrode surface. Upon hybridization with the target DNA, the bioprobe-labeled nanomaterial or enzyme was then recognized for signal amplification. Until now, various nanomaterials such as noble metal nanoparticles, carbon nanotube, graphene, and semiconductor nanoparticles have been fully explored for biosensor fabrication (Swain et al., 2008; Wu et al., 2009; Nie et al., 2012; Benvidi et al., 2015; Jahanbani and Benvidi, 2016; Baluta et al., 2018; Yan et al., 2018; Yu et al., 2019). However, the careful and tedious modification, control, or labeling of biomolecules or reporters onto nanomaterials increases the assay complexity. Thus, development of simple and effective signal amplification means for electrochemical nucleic acid detection is still in high demand.

Herein a simple and sensitive electrochemical nucleic acid biosensor was developed by coupling a zirconia-reduced graphene oxide-thionine (ZrO_2_-rGO-Thi) nanocomposite and exonuclease I (Exo I)-assisted cleavage. The ZrO_2_-rGO nanocomposite was obtained by a one-step hydrothermal synthesis method. Then, a large amount of thionine molecules were adsorbed onto the rGO surface, *via* π-π stacking, as an excellent electrochemical probe. Graphene or graphene oxide-based two-dimensional materials have been widely explored for application in biosensor and biomedicine, owing to their fascinating electronic, thermal, mechanical, and chemical properties (Dong et al., 2012; Chen et al., 2016; Cao et al., 2018; Campos et al., 2019). The obtained ZrO_2_-rGO-Thi nanocomposite was used as an integral multifunctional sensing element for DNA recognition, signal amplification, and reporting. The ZrO_2_ could recognize the phosphate group on the DNA skeleton *via* a multicoordinative interaction. The adsorbed abundant thionine molecules onto the ZrO_2_-rGO nanocomposite are responsible for signal amplification and reporting toward a DNA recognition event. The unique electronic properties of rGO would be also beneficial for improved electrochemical response. Furthermore, it avoids the relatively complex modification, control, or labeling procedures for most of the nanomaterial-based signal amplification strategies. Considered that the ZrO_2_-rGO-Thi nanocomposite may also interact with probe DNA immobilized electrode, a simple Exo I-assisted cleavage was further combined to achieve the discrimination of hybridized from unhybridized DNA and contribute to background reduction. The target DNA related with P53 gene was sensitively analyzed. The P53 gene has been well-known as the guardian of the genome that can code and express p53 protein to suppress the malignant transformation of cells. The p53 gene has been associated with the occurrence of many human tumors such as liver, breast, bladder, and stomach cancers and so on (Hasanzadeh and Shadjou, 2017; Shen et al., 2018). Therefore, the current electrochemical nucleic acid biosensing strategy not only would pave a new avenue for biosensor fabrication by nanomaterials but also is anticipated to show potential for applications in bioanalysis and medical diagnosis.

## Experimental Section

### Chemicals and Reagents

Graphene oxide (GO) was provided by Shanghai TanYuanHuigu New Material Technology Co., Ltd (Shanghai, China). Tris(2-carboxyethyl) phosphine hydrochloride (TCEP), 6-mercaptohexanol (MCH), ZrOCl_2_·8H_2_O, and thionine were purchased from Sigma-Aldrich (St. Louis, MO, USA). Exonuclease I (Exo I) and 10 × Exo I buffer (pH 9.5, 670 mM glycine-KOH, 67 mM MgCl_2_, and 100 mM 2-thioethanol) were obtained from New England Biolabs, Inc. (Ipswich, MA, USA). Fetal calf serum was obtained from Dingguo Biotech Co., Ltd. (Beijing, China). Human serum samples from healthy adults were kindly provided by the Qingdao Central Hospital (Qingdao, China), and informed consent was obtained from all human subjects. The other chemicals were purchased from Shanghai Chemical Reagents (Shanghai, China). The high-performance liquid chromatography-purified DNA sequences were synthesized by Sangon Biotech. Co., Ltd. (Shanghai, China) with the base sequences listed in Table 1. All the solutions were prepared using ultrapure water, with a resistivity of 18.2 MΩ cm.

**Table 1:** The DNA sequences used in the experiment.

**Name**	**Sequence (5^′^ to 3^′^)**
Probe	SH-(CH_2_)_6_-TTT TTT GAG TCT TCC AGT GTG ATG
P53 target	TCA TCA CAC TGG AAG ACT C
1MT	TCA TCA CAC TGG AAG AAT C
2MT	TCA TCA CAC TGG AAG GAT C
NC	GGT CTC TTG ATA GCA CTC A

### Synthesis of ZrO_2_-rGO and ZrO_2_-rGO-Thi NAnocomposites

The ZrO_2_-rGO and the ZrO_2_-rGO-Thi nanocomposites were prepared according to our reported method (Chen et al., 2018). Simply mixed into 20 ml deionized water were 0.004 g of ZrOCl_2_·8H_2_O and 0.004 g of GO, and the mixture was ultrasonically treated for 1 h. The hydrothermal synthesis process for the above mixture was operated at 160°C for 10 h. After the centrifugal collection of the precipitate and freeze-drying, the ZrO_2_-rGO nanocomposite was obtained. The ZrO_2_-rGO-Thi nanocomposites were prepared by mixing 2.5 mg of ZrO_2_-rGO into 10 ml of thionine solution (1 mM) and agitating for 1 h.

### DNA Immobilization on the Electrode Surface

The gold electrodes (2 mm in diameter) were pretreated prior to probe DNA immobilization. The electrodes were polished with 0.3 and 0.05 μm alumina oxide slurries for 5 min, respectively, followed by ultrasonic cleaning in acetone and water to remove residual alumina powder on the electrode surface. Then, the electrodes were electrochemically scanned in 0.5 M H_2_SO_4_ solution, with a potential window ranging from−0.3 to +1.5 V at 100 mV/s for 25 cycles. Finally, the electrodes were rinsed with water and dried with nitrogen. The immobilization of probe DNA on the electrode was performed by immersing into 50 μl of 10 mM Tris-HCl (pH 7.4, 0.1 M NaCl, and 10 mM TCEP) buffer containing 1 μM probe DNA for 4 h at room temperature. The electrode was then rinsed with 20 mM Tris-HCl (pH 7.4, 0.1 M NaCl) buffer solution. After DNA immobilization, the electrode was immersed into 50 μl of MCH solution (1 mM) for 30 min to remove some non-specifically adsorbed DNA strands.

### Target DNA Recognition, Exonuclease I (Exo I) Cleavage, and Binding With ZrO_2_-rGO-Thi Nanocomposite

Target DNA recognition was operated by immersing the probe DNA modified electrode into 50 μl of 10 mM Tris-HCl (pH 7.4, 0.1 M NaCl, and 1 mM MgCl_2_) buffer containing different concentrations of target DNA for 50 min at 37°C. Then, the electrodes were simply rinsed with 20 mM Tris-HCl (pH 7.4, 0.1 M NaCl) buffer solution. After the target DNA recognition, the unhybridized probe DNA was removed by Exo I, which was operated in 1 × exonuclease I reaction buffer (pH 9.5, 67 mM glycine-KOH, 6.7 mM MgCl_2_, and 10 mM 2-thioethanol) containing 5 U of Exo I at 37°C for 60 min. After that, the electrode was thoroughly rinsed with 20 mM Tris-HCl (pH 7.4, 0.1 M NaCl) buffer solution. Then, the electrode was recognized by 0.5 mg/ml ZrO_2_-rGO-Thi nanocomposites for 60 min at room temperature. After the recognition of ZrO_2_-rGO-Thi complex with the electrodes, the electrodes were thoroughly rinsed with 20 mM Tris-HCl (pH 7.4, 0.1 M NaCl) buffer solution to remove some possible non-specifically bounded ZrO_2_-rGO-Thi nanocomposites. Then, the obtained electrode was interrogated for electrochemical measurement.

### Electrochemical Measurements

After binding with the ZrO_2_-rGO-Thi nanocomposite, differential pulse voltammetry (DPV) and cyclic voltammetry (CV) measurements were performed in 10 mM phosphate-buffered saline (PBS; pH 7.4, 0.2 M KNO_3_). The DPV was operated under the following experimental parameters: scanning potential from 0.1 to−0.4 V, amplitude of 50 mV, pulse period of 0.1 s, and sampling width of 16.7 ms. The CV was recorded between 0.2 and−0.6 V, with a scan rate of 100 mV s^−1^. Electrochemical impedance spectroscopy (EIS) was used for biosensor fabrication process characterizations, which was carried out in 10 mM PBS (pH 7.4, 5 mM [Fe(CN)_6_]^3−/4−^ and 1 M KCl), with a scan frequency ranging from 0.1 to 10 kHz and an AC amplitude of 5 mV. Corresponding CV characterizations were also conducted by scanning the potential between−0.1 and 0.6 V at 100 mV s^−1^. Prior to the electrochemical measurement, the electrolyte solution should be purged with high-purity nitrogen for 20 min to avoid interference from oxygen.

### Apparatus

All electrochemical experiments were performed with a CHI 660E electrochemical workstation (CH Instruments, Shanghai, China) at room temperature by using a three-electrode system consisting of a modified gold electrode, a platinum wire auxiliary electrode, and a Ag/AgCl reference electrode. The morphology of the nanocomposite was characterized by a field emission scanning electron microscope (SEM, JSM 7500F, JEOL, Japan) and a transmission electron microscope (TEM, JEM-F200, Japan). Gel images were captured on a FR-980A gel image analysis system (Shanghai, China).

## Results and Discussion

### Detection Principle of the Developed Nucleic Acid Biosensor

**Scheme 1:**
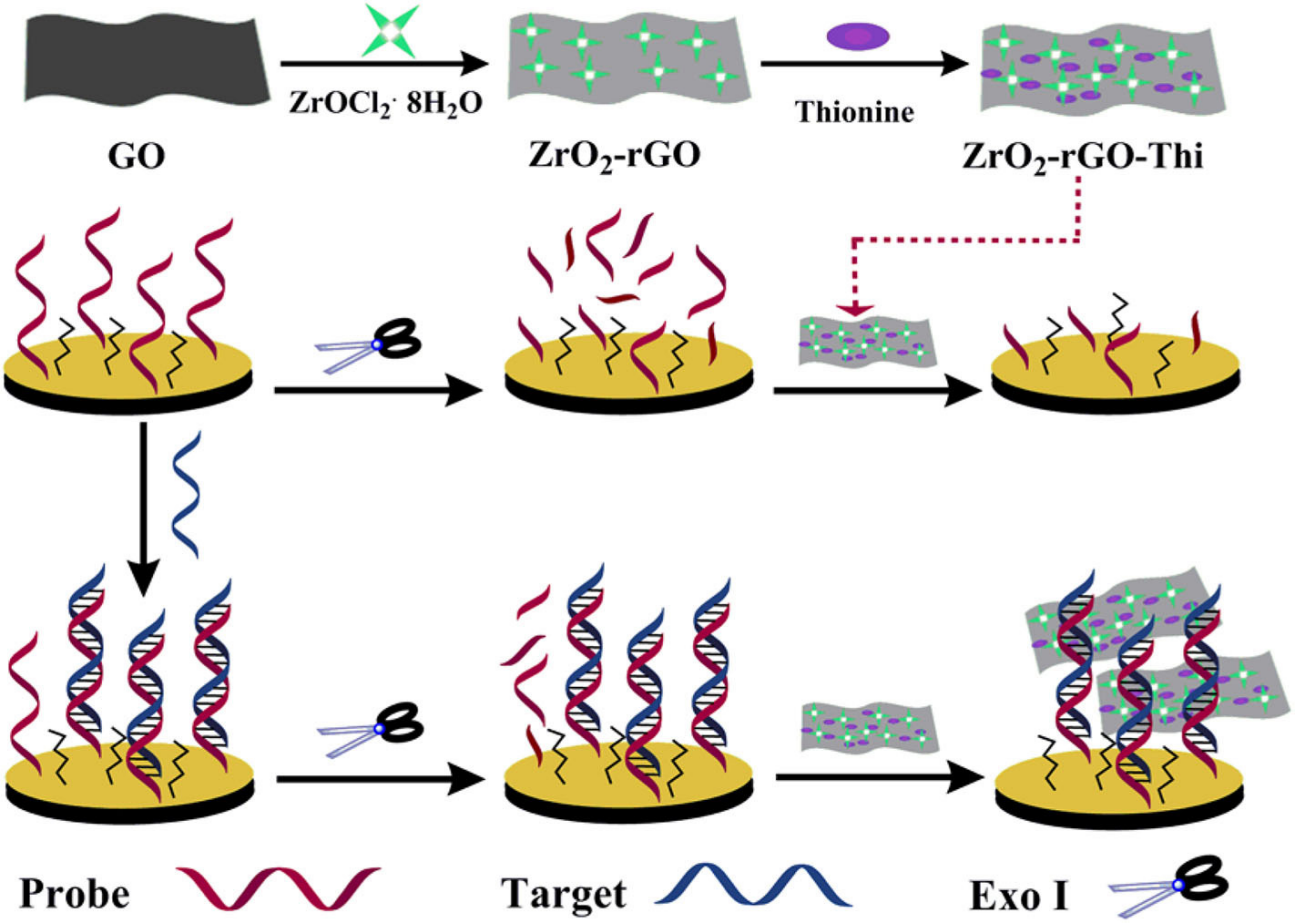
Schematic illustration of electrochemical nucleic acid fabrication by ZrO_2_-rGO-Thi nanocomposite.

The fabrication principle of the electrochemical nucleic acid biosensor by using ZrO_2_-rGO-Thi nanocomposites is illustrated in Scheme 1. The zirconia-reduced graphene oxide (ZrO_2_-rGO) nanocomposites were firstly prepared by a hydrothermal synthesis method, with the use of GO and ZrOCl_2_·8H_2_O as reactants. After ZrOCl_2_ hydrolysis and Zr^4+^ adsorption onto the GO surface, the ZrO_2_ nanoparticles were obtained under hydrothermal conditions. Simultaneously, GO was reduced into rGO to obtain ZrO_2_-rGO nanocomposites. Then, thionine, as a well-characterized electrochemical probe, was conjugated onto the rGO surface *via* π-π interaction to obtain ZrO_2_-rGO-Thi nanocomposite (Li et al., 2012; Zhou et al., 2016). The obtained ZrO_2_-rGO-Thi nanocomposite was used as an integral sensing element for DNA recognition, signal amplification, and reporting. The ZrO_2_ nanoparticles could recognize DNA *via* its multicoordinative interaction with the phosphate group of the DNA skeleton. The multicoordinative interaction of ZrO_2_ with the phosphate group has been widely employed for the enrichment and the analysis of phosphorylated substrates (Monot et al., 2008; Pang et al., 2011). A large amount of thionine molecules adsorbed onto the rGO surface could provide an amplified signal response toward the recognized DNA information. Since the ZrO_2_-rGO-Thi nanocomposite may interact with both the single-stranded DNA and the double-stranded DNA, thus a simple Exo I-assisted cleavage was adopted to achieve DNA discrimination and background reduction. The probe DNA was modified with a sulfhydryl group at the 5′-terminus. The sulfhydryl group could form an Au–S bond with the gold electrode to achieve probe DNA immobilization. In the presence of the target DNA, it can hybridize with the immobilized probe DNA on the electrode. The DNA hybrids formed could resist the cleavage from Exo I, owing to the formation of the blunt 3′-terminus. However, the unhybridized probe DNA would be digested from 3′ to 5′ by Exo I. Then, the remaining hybridized dsDNA onto the electrode could bind with the ZrO_2_-rGO-Thi nanocomposite *via* the multi-coordination between ZrO_2_ and the phosphate group on the DNA skeleton, generating an amplified electrochemical response related with the target DNA recognition event. In the absence of the target DNA, the immobilized probe DNA would be digested by Exo I, and then the ZrO_2_-rGO-Thi nanocomposite could not be effectively captured onto the electrode for an electrochemical response.

### Characterization of ZrO_2_-rGO-Thi Nanocomposites

**Figure 1:**
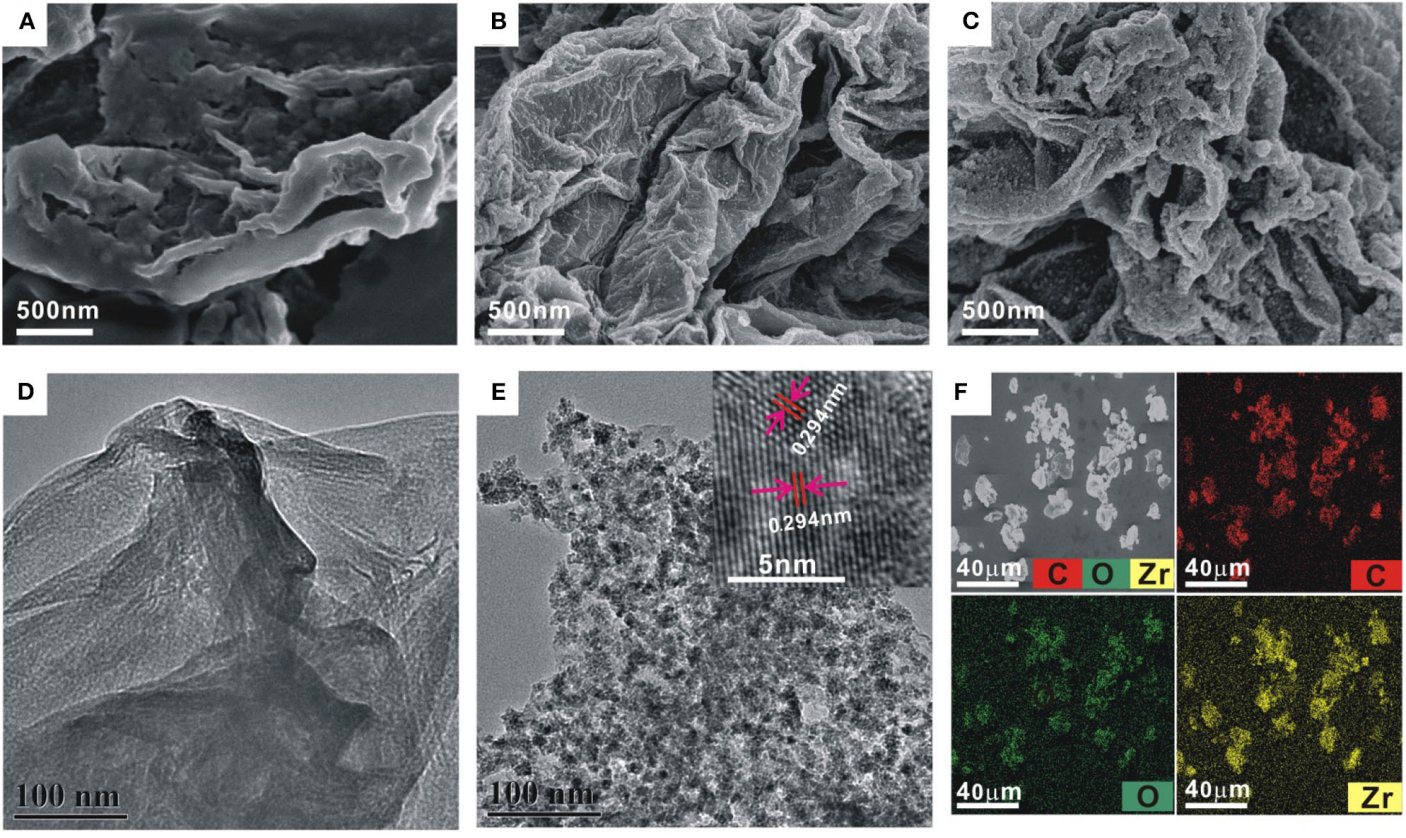
Scanning electron microscope images of GO **(A)** and ZrO_2_-rGO nanocomposite prepared by GO and ZrOCl_2_·8H_2_O with a mass ratio of 1:1 **(B)** and 1:5 **(C)**, respectively. Transmission electron microscope (TEM) images of GO **(D)** and ZrO_2_-rGO nanocomposite prepared by a mass ratio of 1:1 between GO and ZrOCl_2_·8H_2_O **(E)**. The inset in **(E)** shows the high-resolution TEM image of ZrO_2_-rGO nanocomposite. The corresponding elemental mapping images of C, O, and Zr for ZrO_2_-rGO nanocomposite **(F)**.

The morphology of the GO and ZrO_2_-rGO nanocomposites was characterized by SEM and TEM. GO exhibited a typical folded morphology at the edges, confirming the layered structure of GO (Figures 1A,D). After the hydrothermal process for the mixed GO and ZrOCl_2_·8H_2_O (1:1), it could be clearly seen that the ZrO_2_ nanoparticles were uniformly grown on the rGO surface (Figures 1B,E). The interplanar spacing of a single crystalline ZrO_2_ nanoparticle was measured as about 0.294 nm, suggesting a monoclinic phase of the ZrO_2_ nanoparticles (inset in Figure 1E). With the increase of mass ratio between GO and ZrOCl_2_·8H_2_O (1:5), the density of the ZrO_2_ nanoparticles onto the rGO surface was significantly increased (Figure 1C). It could be further seen from the elemental mapping images of ZrO_2_-rGO (Figure 1F) that C, O, and Zr elements were uniformly distributed throughout the nanocomposite. More detailed experimental characterizations toward the ZrO_2_-rGO and the ZrO_2_-rGO-Thi nanocomposites could be referred to our previous work (Chen et al., 2018).

### Electrochemical Characterization of Fabricated DNA Biosensor

**Figure 2:**
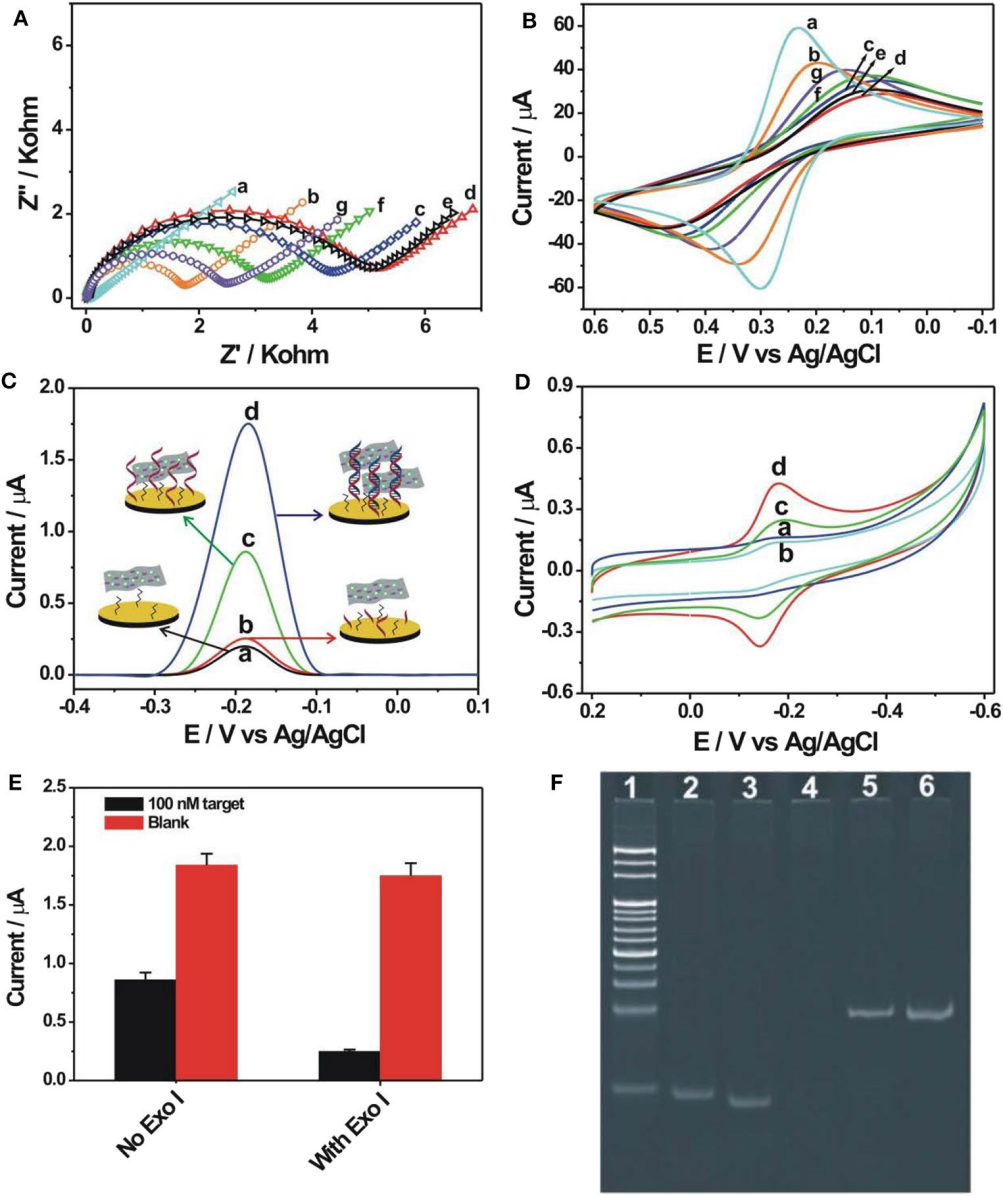
Electrochemical impedance spectroscopy **(A)** and cyclic voltammetry **(B)** recorded into 10 mM phosphate-buffered saline {PBS; pH 7.4, 5 mM [Fe(CN)_6_]^3−/4−^, 1 M KCl} for the different modified electrodes: (a) bare gold electrode, (b) 1.0 μM probe DNA modified electrode, (c) after 6-mercaptohexanol (MCH) blocking, (d) after hybridization with 100 nM target DNA, (e) Exo I cleavage toward (d), (f) after binding with 0.5 mg/ml ZrO_2_-rGO-Thi nanocomposite, and (g) Exo I cleavage toward the probe DNA and MCH modified electrode. Differential pulse voltammetry **(C)** and cyclic voltammetry **(D)** recorded into 10 mM PBS (pH 7.4, 0.2 M KNO_3_) after binding of ZrO_2_-rGO-Thi nanocomposite with different electrodes: (a) MCH-only modified electrode, (c) probe DNA and MCH modified electrode, (b) Exo I cleavage toward probe DNA and MCH modified electrode, and (d) Exo I cleavage toward the hybridized electrodes. The employed amounts for target DNA, Exo I, and ZrO_2_-rGO-Thi nanocomposite were 100 nM, 10 U, and 0.5 mg/ml, respectively. The reaction time for DNA hybridization and Exo I cleavage was 60 min for both. **(E)** Electrochemical response comparison toward blank and 100 nM target DNA in the absence and in the presence of 10 U Exo I. **(F)** Non-denaturing polyacrylamide gel electrophoresis for different DNA sequences: DNA marker (lane 1), 10 μM probe DNA (lane 2), 10 μM target (lane 3), and 1 μM probe DNA after cleavage by 10 U Exo I (lane 4); DNA hybrids by 1 μM probe DNA and 1 μM target DNA (lane 5); and DNA hybrids after cleavage by 10 U Exo I (lane 6).

The EIS was firstly used to follow the nucleic acid biosensor fabrication process by using 5 mM [Fe(CN)_6_]^3−/4−^ as an electrochemical indicator. The corresponding experimental results are shown in Figure 2A. In the EIS curves, a semi-circle diameter represents the corresponding charge transfer resistance (Rct) information of [Fe(CN)_6_]^3−/4−^ toward the electrode surface, which would be easily influenced by the differently modified electrode. The bare gold electrode showed an almost straight line with a Rct value of only 15 Ω (curve a), indicating a rapid charge transfer process of [Fe(CN)_6_]^3−/4−^ toward the electrode surface. When the probe DNA was immobilized onto the electrode, the introduced negative charge by the probe DNA increased the electrostatic repellence toward [Fe(CN)_6_]^3−/4−^, resulting into an increased Rct value of 1,698 Ω (curve b). After blocking with MCH, the Rct value was significantly increased to be about 4,381 Ω (curve c), indicating that the MCH monolayer occupied the vacancy of the electrode surface and evidently inhibited the electron transfer of [Fe(CN)_6_]^3−/4−^ toward the electrode surface. After hybridization with the target DNA (100 nM), the Rct value was further increased to 5,200 Ω (curve d), owing to the introduction of more negative charges by the hybridized target DNA. For the hybridized electrode, the Exo I treatment hardly induced the change of Rct value (5,140 Ω, curve e), suggesting the resistance of the DNA hybrids with the blunt 3′-end against the digestion from Exo I. Then, after the ZrO_2_-rGO-Thi nanocomposites' binding onto the DNA modified electrode, the Rct value was decreased to 3,212 Ω (curve f). It was speculated that some of the bound ZrO_2_-rGO-Thi nanocomposites may be close enough to the electrode surface for the enhanced electron transfer activity. In the absence of the target DNA, after the treatment of the probe DNA modified electrode by Exo I, the Rct value was evidently decreased to 2,500 Ω (curve g) compared with that of the probe DNA and the MCH modified electrode, suggesting the effective digestion of unhybridized probe DNA by Exo I for background reduction.

The corresponding cyclic voltammetric characterizations for the differently modified electrodes are shown in Figure 2B. After probe DNA immobilization (curve b), MCH assembly (curve c), and hybridization with the target DNA (curve d), the redox peak current of [Fe(CN)_6_]^3−/4−^ decreased and the peak-to-peak potential increased sequentially compared with that of the bare gold electrode (curve a), suggesting that the stepwise assembly inhibited the diffusion and the electron transfer of [Fe(CN)_6_]^3−/4−^ toward the electrode surface. The binding of the ZrO_2_-rGO-Thi nanocomposites onto the hybridized electrode could evidently improve the electron transfer performance of [Fe(CN)_6_]^3−/4−^ (curve f). Also, the hybridized DNA could resist the digestion from Exo I cleavage, with almost no change of electrochemical responses of [Fe(CN)_6_]^3−/4−^ compared with that in the absence of Exo I (curve e), but the probe DNA modified electrode could be digested by Exo I cleavage and an improvement of electron transfer of [Fe(CN)_6_]^3−/4−^ could be observed compared with that in the absence of Exo I (curve g). The cyclic voltammetric responses for the differently assembled electrodes were basically in accordance with the EIS results, indicating the successful fabrication of an electrochemical nucleic acid biosensor.

We then conducted the detection feasibility of the fabricated biosensor toward the target DNA by using DPV and CV measurements based on the electrochemical responses of the ZrO_2_-rGO-Thi nanocomposites. The results are shown in Figures 2C,D. It could be seen that, in the presence of 100 nM target DNA, a distinct DPV response of thionine was observed, at a reduction potential of−0.185 V, for the hybridized electrode after Exo I treatment (curve d in Figure 2C). Also, a pair of large redox peaks of thionine, with oxidation and reduction potentials of−0.14 and−0.18 mV, respectively, was obtained in the CV results (curve d in Figure 2D). Thus, the ZrO_2_-rGO-Thi nanocomposites have been effectively combined with the hybridized DNA for an electrochemical response. For the probe DNA modified electrode with no Exo I treatment, an obvious electrochemical response could also be observed (curve c in Figures 2C,D). Thus, the ZrO_2_-rGO-Thi nanocomposites could also be conjugated with the probe DNA modified electrode *via* the multicoordinative interaction for an electrochemical response. However, after Exo I treatment toward the probe DNA modified electrode, only a weak background response could be obtained (curve b in Figures 2C,D). Such a background response was only slightly higher than that of the MCH modified electrode (curve a in Figures 2C,D). Furthermore, an electrochemical response comparison by using Exo I cleavage or not is shown in Figure 2E. The signal-to-background ratio in the presence of Exo I was about 7, but it was only about 2.14 in the absence of Exo I for the detection of 100 nM target DNA. Thus, Exo I could digest the unhybridized probe DNA for background reduction, and the DNA hybrids could resist the cleavage against Exo I and be captured by the ZrO_2_-rGO-Thi nanocomposite for an amplified electrochemical response related with target DNA recognition information. Exo I cleavage toward the probe DNA, but not DNA hybrids, was also confirmed by gel electrophoresis experiments. It could be seen from Figure 2F that the probe DNA (lane 2) and the target DNA (lane 3) showed the corresponding migration bands. The hybridization of the probe DNA and the target DNA showed the band with a lower migration rate (lane 5). After the Exo I treatment, the band related with the probe DNA disappeared (lane 4), suggesting the cleavage of probe DNA by Exo I, but the DNA hybrids with the blunt 3′-end could not be digested by Exo I, and the band related with the DNA hybrids could be still clearly observed (lane 6).

### Optimization of Experimental Conditions

**Figure 3:**
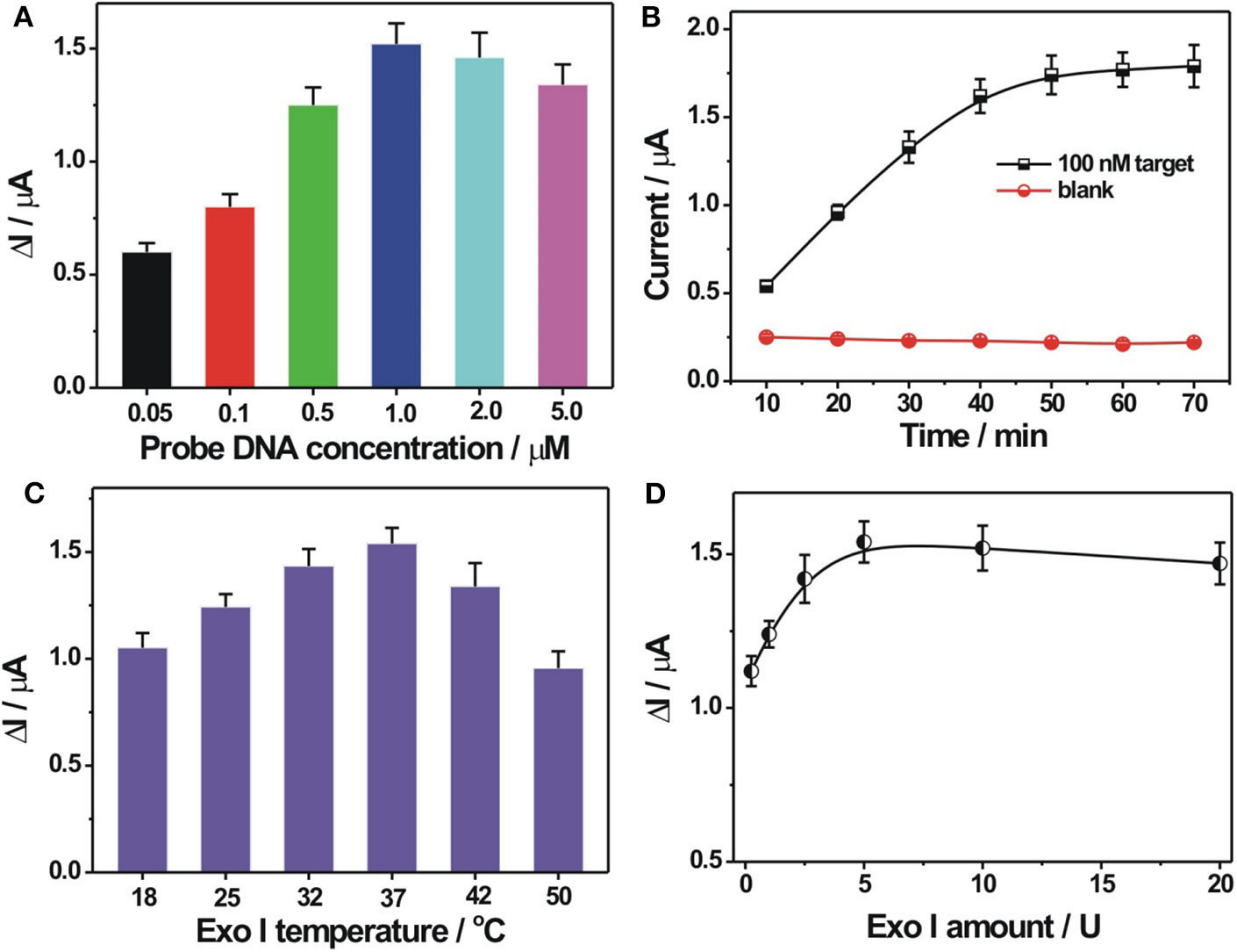
**(A)** Optimization of probe DNA immobilization concentration. The concentration of the probe DNA used was 0.05, 0.1, 0.5, 1.0, 2.0, and 5.0 μ?. **(B)** Effect of hybridization time on the electrochemical responses toward 0 and 100 nM target DNA. **(C)** Optimization of reaction temperature for Exo I cleavage. **(D)** Optimization of Exo I amount. Δ*I* indicates the DPV current difference in the presence and in the absence of target DNA. Error bars, SD, *n* = 3.

In order to demonstrate the best detection capability of the developed biosensor, some critical experimental parameters were optimized, including the immobilization concentration of probe DNA, reaction time, reaction temperature, and amount of Exo I. A proper immobilization concentration of probe DNA would benefit for DNA hybridization recognition and then for electrochemical response. It could be seen from Figure 3A that the DPV current change Δ*I* (Δ*I* = *I*- *I*_0_, *I*, and *I*_0_ represent the current response in the presence and in the absence of the target DNA, respectively), increased with increasing probe DNA immobilization concentrations. It could be easily explained that a higher concentration of probe DNA would increase the assembly amount of probe DNA on the electrode to generate more amounts of DNA hybrids on the electrode for a larger electrochemical response. However, with further increasing probe DNA concentration over 1 μM, the Δ*I* decreased gradually. Since the background response in the absence of the target DNA changed slightly at different probe DNA concentrations, the decreased Δ*I* value obtained at a higher probe DNA concentration may be caused by the decreased recognition efficiency at a higher probe DNA concentration or coverage. Thus, 1 μM was selected as the optimized value for the immobilization concentration of the probe DNA. The surface density of the probe DNA on the electrode was further calculated by the chronocoulometry method with the use of ${\text{Ru}({\text{NH}}_{3})}_{6}^{3+}$ as a redox indicator (Steel et al., 1998). It was 3.27 ± 0.18 × 10^12^ molecules/cm^2^ at an immobilization probe concentration of 1 μM. The hybridization time was also optimized and is shown in Figure 3B. It could be seen that the current response toward 100 nM target DNA increased stepwise with hybridization time extension and almost reached the saturation value at 50 min. Also, in the absence of the target DNA, no evident current change was observed at the studied hybridization time range. Thus, the hybridization time of 50 min was recommended for performance determination of the DNA biosensor. The reaction temperatures for Exo I cleavage and Exo I dosage were also optimized and are shown in Figures 3C,D. It could be seen that the best electrochemical response could be obtained at the reaction temperature of 37°C for Exo I cleavage. It could be explained that 37°C was the suitable temperature to maintain Exo I activity. An elevated temperature would be not beneficial for the stability of the DNA hybrids between the target DNA and the probe DNA, inducing a decreased electrochemical response toward the target DNA. The electrochemical response toward the target DNA increased with the employed Exo I amount, and a maximum value could be reached at an amount of 5 U (Figure 3D). A further increase in Exo I amount would induce a slightly weakened electrochemical response, which might be due to the non-specific cleavage of Exo I toward some hybridized DNA for signal response decrease. Thus, the optimized reaction temperature and the Exo I amount chosen were 37°C and 5 U, respectively.

### Detection Performance of the Developed Electrochemical DNA Biosensor

**Figure 4:**
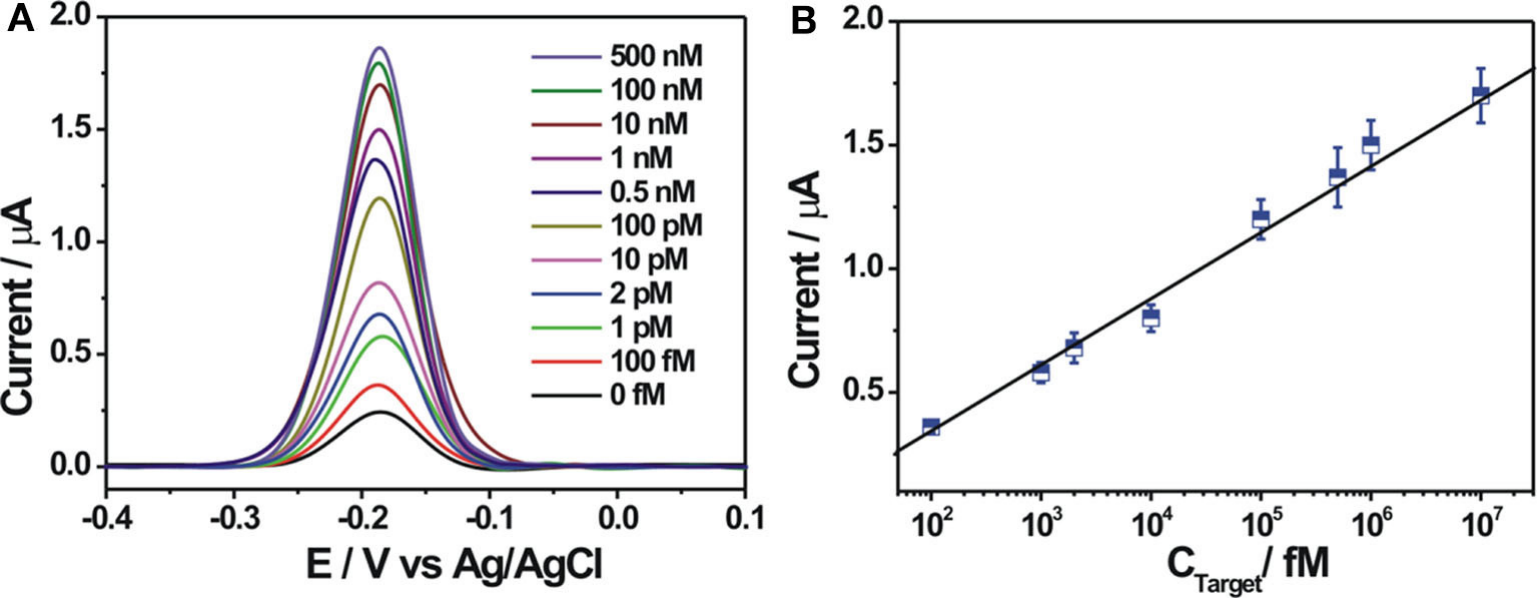
**(A)** Differential pulse voltammetry (DPV) response curves at different concentrations of target DNA (from 0 to 500 nM). **(B)** The linear relationship between the DPV peak current and the logarithmic value of the target DNA concentrations from 100 fM to 10 nM. Error bars, SD, *n* = 3.

The analytical capability of the constructed electrochemical biosensor was investigated by using different concentrations of the target DNA under optimal experimental conditions. As shown in Figure 4A, the electrochemical response signal increased gradually with increasing concentrations of the target DNA, suggesting a concentration-dependent response behavior. A weak background response in the absence of the target DNA could be observed, which might be explained as follows: the immobilized probe DNA may be not completely digested by Exo I, inducing the adsorption of a small amount of ZrO_2_-rGO-Thi nanocomposites for electrochemical response; also, the ZrO_2_-rGO-Thi nanocomposites may be non-specifically adsorbed onto the electrode for electrochemical responses. A linear relationship between the DPV current and the logarithm value of the target DNA concentration, ranging from 100 fM to 10 nM, could be obtained. The corresponding regression equation is expressed as *Y* = −0.1909 + 0.2674 log*X* (*Y* and *X* represent the DPV peak current and the target DNA concentration, respectively), with a correlation coefficient of 0.9943. The detection limit toward the target DNA was calculated as about 24 fM, according to the classic method of LOD = 3β / *k* (β represents the blank response and *k* is the slope of the calibration curve). Such a detection limit was superior or comparable with some reported methods (Table 2), but it only needs a simple Exo I cleavage for background reduction and eliminates the complex DNA designs or too much DNA fragments used by some reported DNA-based signal amplification strategies. The detection reproducibility of the fabricated DNA biosensor toward the target DNA was checked. The relative standard deviations for the detection of 1 pM, 100 pM, and 100 nM were obtained as 6.9, 6.3, and 5.8%, respectively, based on five replicated experiments by different electrodes, suggesting an acceptable detection reproducibility of the fabricated biosensor for the target DNA.

**Table 2:** Comparison of the detection performance of electrochemical DNA biosensors with some reported methods.

**Method**	**Detection limit**	**Strategy**	**Reference**
Colorimetry	10 fM	AuNPs and CHA	(Yun et al., 2016)
Fluorescence	200 pM	CHA and HCR	(Quan et al., 2016)
Fluorescence	50 pM	Dichalcogenide nanosheet	(Zhang et al., 2015)
Fluorescence	100 fM	RCA	(Li et al., 2016)
Electrochemistry	92 fM	Exo III-assisted target recycling	(Tao et al., 2015)
Electrochemistry	36 fM	SDA and DNA Walker	(Wang et al., 2018)
Electrochemistry	2.4 fM	Urchinlike CNT-AuNPs Nanocluster	(Han et al., 2020)
Electrochemistry	10 fM	Triblock polyA DNA probe and enzyme catalysis	(Wang et al., 2019)
Electrochemistry	0.38 pM	Cobalt oxide porous nanocubes	(Kannan et al., 2019)
Electrochemistry	24 fM	rGO-ZrO_2_-Thi and Exo I	This work

*Exo III, exonuclease III; Exo I, exonuclease I; HCR, hybridization chain reaction; CHA, catalytic hairpin assembly; AuNPs, gold nanoparticles; CNT, carbon nanotube*.

### Selectivity and Stability of Fabricated Electrochemical DNA Biosensor

**Figure 5:**
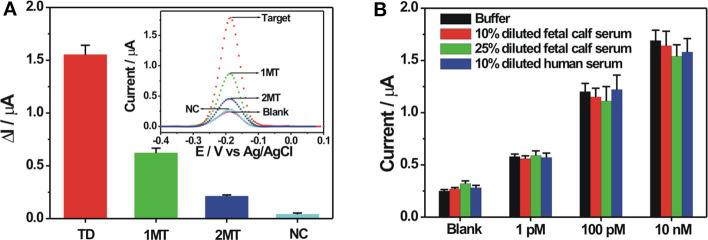
**(A)** Selectivity of the fabricated biosensor toward 100 nM of various DNA sequences including complementary target, single-base mismatched DNA (1 MT), two-base mismatched DNA (2 MT), and non-complementary DNA (NC). The inset shows the corresponding DPV responses toward the different DNA sequences. **(B)** Electrochemical responses of the fabricated biosensor toward three different concentrations of target DNA spiked in buffer, 10 and 25% diluted fetal bovine serum, and 10% diluted human serum. Error bars, SD, *n* = 3.

The selectivity of the proposed electrochemical biosensor was evaluated by using different DNA sequences, including fully complementary target DNA, single-base mismatched target (1MT), two-base mismatched target (2MT), and non-complementary DNA (NC) with the same concentration. As can be seen in Figure 5A, the DPV current response of NC was almost the same with the blank solution. The mismatched DNA showed the decreased electrochemical responses compared with the complementary target DNA, owing to the decreased hybridization stability with the probe DNA. The DPV responses for 1MT and 2MT were about 40 and 13.5% of that for the fully complementary target DNA, respectively, demonstrating the potential for base mutation analysis. The stability of the probe DNA modified electrode was then investigated. After its storage at 4°C for 14 days, the electrochemical response toward 100 nM target DNA could still remain over 90% of its original response, suggesting the robust stability of the fabricated biosensor. To demonstrate the applicative potential of the fabricated DNA biosensor in the relatively complex biological samples, detections of target DNA spiked in 10 and 25% diluted fetal calf serum and 10% diluted human serum were conducted. It could be seen in Figure 5B that the diluted serum could exert some influences on the background and the signal responses, but the electrochemical responses were still dependent on the spiked target DNA concentration in the diluted serum. The background responses in the diluted serum are slightly larger than that in the buffer. It might be due to the non-specific adsorption of some biological molecules in the serum on the electrode, further inducing the possible adsorption of the ZrO_2_-rGO-Thi nanocomposite for increased background response. The signal response in the diluted serum was mostly lower than that in the buffer. It might be explained that the non-specific adsorbed biological molecules on the electrode influenced the DNA hybridization recognition to some extent for the observed signal decrease. These results suggested an applicative potential of our fabricated DNA biosensor in relatively complex biological matrices.

## Conclusions

In conclusion, a simple electrochemical biosensor was developed for sensitive DNA detection by coupling the ZrO_2_-rGO-Thi nanocomposite and Exo I-assisted cleavage. The ZrO_2_-rGO-Thi nanocomposite was explored as an integral sensing nanomaterial, including DNA recognition (multi-coordinative interaction of ZrO_2_ with the phosphate group of the DNA skeleton), signal amplification and reporting (abundant thionines onto the rGO surface for direct electrochemical response), and excellent conductivity of rGO. With the aid of Exo I-based cleavage for background reduction, the sensitive and the selective detection of target DNA related with the P53 gene could be achieved with a wide linear detection range (100 fM to 10 nM) and a low detection limit of 24 fM. Although the detection limit by the current biosensor is still higher compared with those most sensitive methods reported, it does not involve complex DNA designs or operations or too much DNA fragments for signal amplification. It can also be applied for DNA detection in a relatively complex biological matrix such as serum. Therefore, it offers a simple but effective biosensor fabrication strategy and is anticipated to show potential for applications in bioanalysis and medical diagnosis.

## Data Availability Statement

All datasets generated for this study are included in the article/supplementary material.

## Author Contributions

ZC and SL: designed the work and wrote the manuscript. ZC, XL, DL, FL, and LW: carried out the experiments. ZC and DL: performed the statistical analysis. FL and SL: revised and edited the manuscript. All authors reviewed the manuscript and have agreed to its publication.

## Conflict of Interest

The authors declare that the research was conducted in the absence of any commercial or financial relationships that could be construed as a potential conflict of interest.

## References

[B1] Asiello P. J. Baeumner A. J. (2011). Miniaturized isothermal nucleic acid amplification, a review. Lab. Chip. 11, 1420–1430. 10.1039/c0lc00666a 21387067

[B2] Baluta S. Lesiak A. Cabaj J. (2018). Graphene quantum dots-based electrochemical biosensor for catecholamine neurotransmitters detection. Electroanal 30, 1791–1800. 10.1002/elan.201700825

[B3] Benvidi A. Dehghani Firouzabadi A. Dehghan Tezerjani M. Moshtaghiun S. M. Mazloum-Ardakani M. Ansarin A. (2015). A highly sensitive and selective electrochemical DNA biosensor to diagnose breast cancer. J. Electroanal. Chem. 750, 57–64. 10.1016/j.jelechem.2015.05.002

[B4] Campos R. Borme J. Guerreiro J. R. Machado G Jr Cerqueira M. F. Petrovykh D. Y.. (2019). Attomolar label-free detection of DNA hybridization with electrolyte-gated graphene field-effect transistors. ACS Sens. 4, 286–293. 10.1021/acssensors.8b00344 30672282

[B5] Cao C. Jin R. Wei H. Yang W. Goldys E. M. Hutchinson M. R.. (2018). Graphene oxide based recyclable *in vivo* device for amperometric monitoring of interferon-γ in inflammatory mice. ACS Appl. Mater. Interfaces 10, 33078–33087. 10.1021/acsami.8b13518 30199621

[B6] Chen J. Y. Liu Z. J. Wang X. W. Ye C. L. Zheng Y. J. Peng H. P.. (2019a). Ultrasensitive electrochemical biosensor developed by probe lengthening for detection of genomic DNA in human serum. Anal. Chem. 91, 4552–4558. 10.1021/acs.analchem.8b05692 30838849

[B7] Chen L. Song L. Zhang Y. Wang P. Xiao Z. Guo Y.. (2016). Nitrogen and sulfur codoped reduced graphene oxide as a general platform for rapid and sensitive fluorescent detection of biological species. ACS Appl. Mater. Interfaces 8, 11255–11261. 10.1021/acsami.6b01030 27089122

[B8] Chen X. Liu Y. Xu L. Wang Y. Li R. Sun P.. (2019b). Jungle on the electrode: a target-induced enzyme-free and label-free biosensor. Anal. Chem. 91, 13712–13719. 10.1021/acs.analchem.9b03004 31588727

[B9] Chen Z. Liu Y. Hao L. Zhu Z. Li F. Liu S. (2018). Reduced graphene oxide-zirconium dioxide–thionine nanocomposite integrating recognition, amplification, and signaling for an electrochemical assay of protein kinase activity and inhibitor screening. ACS Appl. Bio. Mater. 1, 1557–1565. 10.1021/acsabm.8b00451 34996206

[B10] Debouck C. Goodfellow P. N. (1999). DNA microarrays in drug discovery and development. Nat. Genet. 21, 48–50. 10.1038/4475 9915501

[B11] Ding L. Zhang L. Yang H. Liu H. Ge S. Yu J. (2018). Electrochemical biosensor for p53 gene based on HRP-mimicking DNAzyme-catalyzed deposition of polyaniline coupled with hybridization chain reaction. Sens. Actuators B Chem. 268, 210–216. 10.1016/j.snb.2018.04.126

[B12] Ding X. Cheng W. Li Y. Wu J. Li X. Cheng Q.. (2017). An enzyme-free surface plasmon resonance biosensing strategy for detection of DNA and small molecule based on nonlinear hybridization chain reaction. Biosens. Bioelectr. 87, 345–351. 10.1016/j.bios.2016.08.077 27587359

[B13] Dong H. Zhang J. Ju H. Lu H. Wang S. Jin S.. (2012). Highly sensitive multiple microRNA detection based on fluorescence quenching of graphene oxide and isothermal strand-displacement polymerase reaction. Anal. Chem. 84, 4587–4593. 10.1021/ac300721u 22510208

[B14] Feng Q. M. Guo Y. H. Xu J. J. Chen H. Y. (2017). Self-assembled DNA tetrahedral scaffolds for the construction of electrochemiluminescence biosensor with programmable DNA cyclic amplification. ACS Appl. Mater. Interfaces 9, 17637–17644. 10.1021/acsami.7b04553 28471159

[B15] Han S. Liu W. Zheng M. Wang R. (2020). Label-free and ultrasensitive electrochemical DNA biosensor based on urchinlike carbon nanotube-gold nanoparticle nanoclusters. Anal. Chem. 92, 4780–4787. 10.1021/acs.analchem.9b03520 32054266

[B16] Hasanzadeh M. Shadjou N. (2017). (Nano)-materials and methods of signal enhancement for genosensing of p53 tumor suppressor protein: novel research overview. Mater. Sci. Eng. C 76, 1424–1439. 10.1016/j.msec.2017.02.038 28482509

[B17] Hu Y. Xu X. Liu Q. Wang L. Lin Z. Chen G. (2014). Ultrasensitive electrochemical biosensor for detection of DNA from Bacillus subtilis by coupling target-induced strand displacement and nicking endonuclease signal amplification. Anal. Chem. 86, 8785–8790. 10.1021/ac502008k 25112918

[B18] Jahanbani S. Benvidi A. (2016). A novel electrochemical DNA biosensor based on a modified magnetic bar carbon paste electrode with Fe_3_O_4_ NPs-reduced graphene oxide/PANHS nanocomposite. Mater. Sci. Eng. C 68, 1–8. 10.1016/j.msec.2016.05.056 27523989

[B19] Kannan P. Subramanian P. Maiyalagan T. Jiang Z. (2019). Cobalt oxide porous nanocubes-based electrochemical immunobiosensing of hepatitis B virus DNA in blood serum and urine samples. Anal. Chem. 91, 5824–5833. 10.1021/acs.analchem.9b00153 30917656

[B20] Karunanayake Mudiyanselage A. P. Yu Q. Leon-Duque M. A. Zhao B. Wu R. You M. (2018). Genetically encoded catalytic hairpin assembly for sensitive RNA imaging in live cells. J. Am. Chem. Soc.140, 8739–8745. 10.1021/jacs.8b03956 29944357 PMC6201751

[B21] Li H. Xu J. Wang Z. Wu Z. S. Jia L. (2016). Increasingly branched rolling circle amplification for the cancer gene detection. Biosens. Bioelectr. 86, 1067–1073. 10.1016/j.bios.2016.07.095 27569300

[B22] Li J. Fu W. Bao J. Wang Z. Dai Z. (2018). Fluorescence regulation of copper nanoclusters via DNA template manipulation toward design of a high signal-to-noise ratio biosensor. ACS Appl. Mater. Interfaces 10, 6965–6971. 10.1021/acsami.7b19055 29363949

[B23] Li W. Wu P. Zhang H. Cai C. (2012). Signal amplification of graphene oxide combining with restriction endonuclease for site-specific determination of DNA methylation and assay of methyltransferase activity. Anal. Chem. 84, 7583–7590. 10.1021/ac301990f 22882077

[B24] Liu G. Wan Y. Gau V. Zhang J. Wang L. Song S.. (2008). An enzyme-based E-DNA sensor for sequence-specific detection of femtomolar DNA targets. J. Am. Chem. Soc. 130, 6820–6825. 10.1021/ja800554t 18459781

[B25] Liu S. Fang L. Wang Y. Wang L. (2017). Universal dynamic DNA assembly-programmed surface hybridization effect for single-step, reusable, and amplified electrochemical nucleic acid biosensing. Anal. Chem. 89, 3108–3115. 10.1021/acs.analchem.6b04871 28194961

[B26] Lv Y. Cui L. Peng R. Zhao Z. Qiu L. Chen H.. (2015). Entropy beacon: a hairpin-free DNA amplification strategy for efficient detection of nucleic acids. Anal. Chem. 87, 11714–11720. 10.1021/acs.analchem.5b02654 26505212 PMC4898272

[B27] Monot J. Petit M. Lane S. M. Guisle I. Léger J. Tellier C.. (2008). Towards zirconium phosphonate-based microarrays for probing DNA-protein interactions: critical influence of the location of the probe anchoring groups. J. Am. Chem. Soc. 130, 6243–6251. 10.1021/ja711427q 18407629

[B28] Nie H. Yang Z. Huang S. Wu Z. Wang H. Yu R.. (2012). DNA-wrapped carbon nanotubes as sensitive electrochemical labels in controlled-assembly-mediated signal transduction for the detection of sequence-specific DNA. Small 8, 1407–1414. 10.1002/smll.201102071 22392696

[B29] Pang H. Lu Q. Gao F. (2011). Graphene oxide induced growth of one-dimensional fusiform zirconia nanostructures for highly selective capture of phosphopeptides. Chem. Commun. 47, 11772–11774. 10.1039/c1cc14618a 21952079

[B30] Quan K. Huang J. Yang X. Yang Y. Ying L. Wang H.. (2016). Powerful amplification cascades of FRET-based two-layer nonenzymatic nucleic acid circuits. Anal. Chem. 88, 5857–5864. 10.1021/acs.analchem.6b00609 27142084

[B31] Shen Z. F. Li F. Jiang Y. F. Chen C. Xu H. Li C. C.. (2018). Palindromic molecule beacon-based cascade amplification for colorimetric detection of cancer genes. Anal. Chem. 90, 3335–3340. 10.1021/acs.analchem.7b04895 29411603

[B32] Shi X. M. Fan G. C. Shen Q. Zhu J. J. (2016). Photoelectrochemical DNA biosensor based on dual-signal amplification strategy integrating inorganic–organic nanocomposites sensitization with λ-exonuclease-assisted target recycling. ACS Appl. Mater. Interfaces 8, 35091–35098. 10.1021/acsami.6b14466 27983802

[B33] Steel A. B. Herne T. M. Tarlov M. J. (1998). Electrochemical quantitation of DNA immobilized on gold. Anal. Chem. 70, 4670–4677. 10.1021/ac980037q 9844566

[B34] Swain M. D. Octain J. Benson D. E. (2008). Unimolecular, soluble semiconductor nanoparticle-based biosensors for thrombin using charge/electron transfer. Bioconjugate Chem. 19, 2520–2526. 10.1021/bc8003952 19053236

[B35] Tan Y. Wei X. Zhao M. Qiu B. Guo L. Lin Z.. (2015). Ultraselective homogeneous electrochemical biosensor for DNA species related to oral cancer based on nicking endonuclease assisted target recycling amplification. Anal Chem. 87, 9204–9208. 10.1021/acs.analchem.5b01470 26295334

[B36] Tao C. Yan Y. Xiang H. Zhu D. Cheng W. Ju H.. (2015). A new mode for highly sensitive and specific detection of DNA based on exonuclease III-assisted target recycling amplification and mismatched catalytic hairpin assembly. Chem Commun. 51, 4220–4222. 10.1039/C5CC00385G 25669420

[B37] Wang K. He M. Q. Zhai F. H. Wang J. He R. H. Yu Y. L. (2018). Autonomous DNA nanomachine based on cascade amplification of strand displacement and DNA walker for detection of multiple DNAs. Biosens. Bioelectr. 105, 159–165. 10.1016/j.bios.2018.01.044 29412940

[B38] Wang L. Wen Y. Yang X. Xu L. Liang W. Zhu Y.. (2019). Ultrasensitive electrochemical DNA biosensor based on a label-free assembling strategy using a triblock polyA DNA probe. Anal. Chem. 91, 16002–16009. 10.1021/acs.analchem.9b04757 31746200

[B39] Wang Q. Yang C. Xiang Y. Yuan R. Chai Y. (2014). Dual amplified and ultrasensitive electrochemical detection of mutant DNA biomarkers based on nuclease-assisted target recycling and rolling circle amplifications. Biosens. Bioelectr. 55, 266–271. 10.1016/j.bios.2013.12.034 24393655

[B40] Wang Y. Bai X. Wen W. Zhang X. Wang S. (2015). Ultrasensitive electrochemical biosensor for HIV gene detection based on graphene stabilized gold nanoclusters with exonuclease amplification. ACS Appl. Mater. Interfaces 7, 18872–18879. 10.1021/acsami.5b05857 26252625

[B41] Wu Z. Zhen Z. Jiang J. H. Shen G. L. Yu R. Q. (2009). Terminal protection of small-molecule-linked DNA for sensitive electrochemical detection of protein binding via selective carbon nanotube assembly. J. Am. Chem. Soc. 131, 12325–12332. 10.1021/ja9038054 19655753

[B42] Xu J. Wu Z. S. Shen W. Le J. Zheng T. Li H.. (2017). Programmable nanoassembly consisting of two hairpin-DNAs for p53 gene determination. Biosens. Bioelectr. 94, 626–631. 10.1016/j.bios.2017.03.052 28371752

[B43] Yan T. Zhu L. Ju H. Lei J. (2018). DNA-walker-induced allosteric switch for tandem signal amplification with palladium nanoparticles/metal-organic framework tags in electrochemical biosensing. Anal. Chem. 90, 14493–14499. 10.1021/acs.analchem.8b04338 30472833

[B44] Yu N. Zhang X. Gao Y. You H. Zhang J. Miao P. (2019). Highly sensitive endotoxin assay combining peptide/graphene oxide and DNA-modified gold nanoparticles. ACS Omega 4, 14312–14316. 10.1021/acsomega.9b02013 31508556 PMC6733170

[B45] Yun W. Jiang J. Cai D. Zhao P. Liao J. Sang G. (2016). Ultrasensitive visual detection of DNA with tunable dynamic range by using unmodified gold nanoparticles and target catalyzed hairpin assembly amplification. Biosens. Bioelectr. 77, 421–427. 10.1016/j.bios.2015.09.065 26448518

[B46] Zhang Y. Zheng B. Zhu C. Zhang X. Tan C. Li H.. (2015). Single-layer transition metal dichalcogenide nanosheet-based nanosensors for rapid, sensitive, and multiplexed detection of DNA. Adv. Mater. 27, 935–939. 10.1002/adma.201404568 25504749

[B47] Zhang Z. Balogh D. Wang F. Sung S. Y. Nechushtai R. Willner I. (2013). Biocatalytic release of an anticancer drug from nucleic-acids-capped mesoporous SiO_2_ using DNA or molecular biomarkers as triggering stimuli. ACS Nano. 7, 8455–8468. 10.1021/nn403772j 23985013

[B48] Zhou Q. Li G. Zhang Y. Zhu M. Wan Y. Shen Y. (2016). Highly selective and sensitive electrochemical immunoassay of Cry1C using nanobody and π-π stacked graphene oxide/thionine assembly. Anal. Chem. 88, 9830–9836. 10.1021/acs.analchem.6b02945 27617345

